# Arylacetamide Deacetylase Is Involved in Vicagrel Bioactivation in Humans

**DOI:** 10.3389/fphar.2017.00846

**Published:** 2017-11-20

**Authors:** Jinfang Jiang, Xiaoyan Chen, Dafang Zhong

**Affiliations:** ^1^State Key Laboratory of Drug Research, Center for Drug Metabolism and Pharmacokinetics Research, Shanghai Institute of Materia Medica, Chinese Academy of Sciences, Shanghai, China; ^2^University of Chinese Academy of Sciences, Beijing, China

**Keywords:** arylacetamide deacetylase, vicagrel, clopidogrel, hydrolytic metabolism

## Abstract

Vicagrel, a structural analog of clopidogrel, is now being developed as a thienopyridine antiplatelet agent in a phase II clinical trial in China. Some studies have shown that vicagrel undergoes complete first-pass metabolism in human intestine, generating the hydrolytic metabolite 2-oxo-clopidogrel via carboxylesterase-2 (CES2) and subsequently the active metabolite H4 via CYP450s. This study aimed to identify hydrolases other than CES2 that are involved in the bioactivation of vicagrel in human intestine. This study is the first to determine that human arylacetamide deacetylase (AADAC) is involved in 2-oxo-clopidogrel production from vicagrel in human intestine. *In vitro* hydrolytic kinetics were determined in human intestine microsomes and recombinant human CES and AADAC. The calculated contribution of CES2 and AADAC to vicagrel hydrolysis was 44.2 and 53.1% in human intestine, respectively. The AADAC-selective inhibitors vinblastine and eserine effectively inhibited vicagrel hydrolysis *in vitro*. In addition to CES2, human intestine AADAC was involved in vicagrel hydrolytic activation before it entered systemic circulation. In addition, simvastatin efficiently inhibited the production of both 2-oxo-clopidogrel and active H4; further clinical trials are needed to determine whether the hydrolytic activation of vicagrel is influenced by coadministration with simvastatin. This study deepens the understanding of the bioactivation and metabolism properties of vicagrel in humans, which can help further understand the bioactivation mechanism of vicagrel and the variations in the treatment responses to vicagrel and clopidogrel.

## Introduction

**FIGURE 1:**
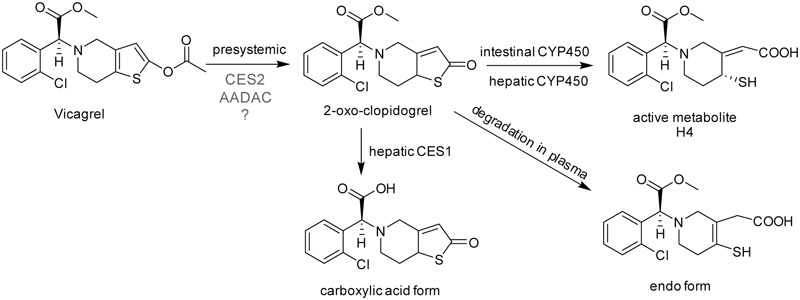
The chemical structures and proposed metabolic pathway of vicagrel in humans. According to a previous report (Qiu et al., 2016), vicagrel was hydrolyzed by CES2. Vicagrel is efficiently hydrolyzed in dog intestine where CES2 is not expressed; thus, the involvement of other enzymes is presumed.

The thiophenopyridine antiplatelet agent clopidogrel is a second-generation platelet ADP receptor antagonist that has become a routine drug for antiplatelet therapy after coronary intervention. Clopidogrel is an inactive prodrug, approximately 85% of clopidogrel is hydrolyzed by CES1 to an inactive carboxylic acid metabolite, while the remaining 15% is oxidized by CYP450s (mainly mediated by CYP2C19) to generate the active thiol metabolite H4 (Hagihara et al., 2009; Farid et al., 2010). H4 (**Figure 1**) is the main active ingredient for clopidogrel, that prevents platelet aggregation by irreversibly inhibiting the platelet receptor P2Y12 (Pereillo et al., 2002). CYP2C19 gene polymorphism affects the treatment response to clopidogrel. Some patients showed low or even no response to clopidogrel treatment (Barragan et al., 2003; Gurbel et al., 2005), which is called “clopidogrel resistance.” In China, the major CYP2C19 genotypes are ^∗^1/^∗^1 and ^∗^1/^∗^2, each of which comprise approximately 43% of the population (Myrand et al., 2008). Approximately 16% of the Chinese population carry two CYP2C19-deficient genes, and become the poor metabolizers of clopidogrel (Kelly et al., 2012). Studies have shown that the probability of cardiovascular adverse events in patients with two CYP2C19-deficient genes (^∗^ 2, ^∗^ 3, ^∗^ 4, or ^∗^ 5) was 3.58 times higher than in patients without mutated genes (Simon et al., 2009).

Because of these clinical defects of clopidogrel, vicagrel was designed to avoid metabolism by liver CYP2C19 and to be metabolized instead by esterases in the intestine and liver, aiming to produce H4 more efficiently and consistently in humans than clopidogrel. Vicagrel is the acetate form of the clopidogrel hydroxylated structure (**Figure 1**). In rats and beagle dogs, the production of 2-oxo-clopidogrel and H4 from vicagrel was about six times and four to six times higher than that of clopidogrel (Qiu et al., 2013), respectively. Vicagrel undergoes complete first-pass metabolism in the intestine to produce 2-oxo-clopidogrel and the subsequent active metabolite H4 by CYP450s in the intestine and liver (Qiu et al., 2014). Formation of 2-oxo-clopidogrel from vicagrel is catalyzed by non-CYP450 enzymes, which eliminates the incidence of “clopidogrel resistance” caused by genetic polymorphisms and increases the proportion of the active metabolite. Vicagrel is a very promising new drug and is currently being evaluated in a phase II clinical trial in China.

The first hydrolysis step is the most important step in vicagrel bioactive pathway. Studies have shown that the enzyme responsible for vicagrel hydrolysis in the intestine is CES2 (Qiu et al., 2014). In addition, Qiu et al. (2016) found that vicagrel was rapidly hydrolyzed in dog intestine; the formation rate of active H4 was higher in dog intestine than in rat and human intestines. To date, no esterase has been found in the intestine of dogs (Berry et al., 2009), which prompted the current investigation into the involvement of enzymes other than CES in vicagrel bioactivation.

Arylacetamide deacetylase (AADAC) is a kind of serine hydrolase that is widely involved in the hydrolysis of drugs. AADAC is mainly expressed in human intestine and liver (Fukami and Yokoi, 2012) and is responsible for the hydrolysis of flutamide (Watanabe et al., 2009), phenacetin (Watanabe et al., 2010), and rifampicin (Nakajima et al., 2011); it is closely associated with the renal failure and liver toxicity that occurs in some patients after taking flutamide or phenacetin (Fukami and Yokoi, 2012). The antiplatelet drug prasugrel also contains an acetyl ester bond in the thiophene ring. Intestinal AADAC and CES2 comediated the hydrolysis of prasugrel to produce a thiolactone metabolite; AADAC contributed approximately 50% to its hydrolysis (Kurokawa et al., 2016). Kurokawa et al. (2016) found that even though CES protein was not expressed in dog intestine, AADAC activity was functionally observed in dog intestine. Vicagrel contains the same acetyl ester bond in the thiophene ring as prasugrel, which confers the substrate specificity of AADAC (Fukami et al., 2015). In addition, vicagrel was rapidly hydrolyzed in dog intestine in a previous study (Qiu et al., 2016). We speculated that AADAC was also involved in the hydrolysis of vicagrel.

To verify our hypothesis, we first investigated the hydrolytic kinetics of vicagrel in HLM, intestinal microsomes, recombinant CES and AADAC enzymes. The contributions of CES2 and AADAC to vicagrel were calculated by the RAF method using procaine and phenacetin as probe substrates. Finally, the effects of various chemical inhibitors were investigated to further confirm our hypothesis.

## Materials and Methods

### Chemicals and Reagents

Vicagrel (99.0% purity), 2-oxo-clopidogrel (99.0% purity), 2-oxo-clopidogrel-d_3_ (98.6% purity), H4 derivative (H4-MP, 95.1% purity) and H4-d_3_ derivative (H4-d_3_-MP, 93.9% purity) were provided by Jiangsu Vcare Pharmatech Co., Ltd. (Jiangsu, China). Fenofibrate, phenacetin, procaine, loperamide, BNPP, and 3-methoxyphenacyl bromide (MPB) were purchased from Sigma–Aldrich (St. Louis, MO, United States). Digitonin, vinblastine, eserine, atorvastatin, and simvastatin were purchased from Dalian Meilun Biology and Technology Co., Ltd. (Dalian, China). HLM and HIM were purchased from BD Gentest (Woburn, MA, United States). Recombinant human CES1 and CES2 were purchased from Cypex Ltd. (Scotland, United Kingdom), and recombinant human AADAC was purchased from CUSABIO Biotech Co., Ltd. (Wuhan, China). Deionized water (18.2 mΩ, TOC ≤ 50 ppb) was purified using a Millipore Milli-Q Gradient Water Purification System (Molsheim, France). All other chemicals were of analytical grade.

### Vicagrel Hydrolase Activity

Vicagrel hydrolase activities in different enzyme sources were determined as follows. The incubation mixture (at a final volume of 0.2 mL) contained 100 mM potassium phosphate buffer (containing 3.2 mM MgCl_2_, pH 7.4) and enzyme sources (HLM, HIM, and recombinant human CES1, CES2, and AADAC). The final concentration of the organic reagent was <1% in the incubation volume. We confirmed that the formation rates of 2-oxo-clopidogrel from vicagrel were linear with respect to protein concentration (HLM and HIM < 0.02 mg/mL; CES1, CES2, and AADAC < 0.03 mg/mL) and incubation time (<5 min). The final conditions were as follows: HLM or HIM: 0.01 mg/mL, 1 min; CES1: 0.01 mg/mL, 2 min; CES2: 0.01 mg/mL, 1 min; and AADAC: 0.015 mg/mL, 2 min. After 5 min of preincubation at 37°C, reactions were initiated by the addition of vicagrel (0.1–100 μM). After incubation, the reactions were terminated by adding equal volumes of ice-cold acetonitrile. Control samples were incubated with thermally inactivated enzymes. We also investigated vicagrel hydrolase activity in DIM, at 0.01 mg/mL and for 1 min. The data were presented as averages of triplicate experiments. Kinetic constants were obtained by fitting experimental data to the Michaelis–Menten equation using non-linear regression, as shown in Equation 1 (Prism 5.0; GraphPad Software Inc., San Diego, CA, United States):

(1) $$v\;=\;V_{\text{max}}\;\times \;S/(K_{\text{m}}\;+\;S)$$

where *v* is the reaction velocity, *V*_max_ is the maximum velocity, *K*_m_ is the Michaelis constant (substrate concentration at 0.5*V*_max_), and *S* is the substrate concentration. *In vitro* CL_int_ was calculated as *V*_max_/*K*_m_.

Fenofibrate, procaine, and phenacetin are specific substrates of CES1, CES2, and AADAC, respectively. Hydrolysis of phenacetin was performed according to previous studies (Watanabe et al., 2010; Fukami et al., 2015) but with some changes. Considering the solubility of the substrates and the ease of detection, we adjusted the concentration of the substrates in the incubation systems. The substrate concentrations of phenacetin, fenofibrate, and procaine were all set to 200 μM. We confirmed that the hydrolytic rates were linear with respect to protein concentrations and incubation time. For phenacetin, the enzyme source concentration was 0.4 mg/mL, and the incubation time was 30 min. For fenofibrate, the enzyme sources concentration was 0.05 mg/mL, and the incubation time was 5 min. For procaine, the enzyme sources concentration was 0.1 mg/mL, and the incubation time was 30 min.

### Inhibition on Vicagrel Hydrolase Activity

To confirm that the esterase isoform is involved in vicagrel hydrolysis in humans, we conducted inhibition studies on HIM, and recombinant CES2 and AADAC using representative esterase inhibitors. BNPP is a non-selective inhibitor of esterases (Watanabe et al., 2009), and digitonin and loperamide specifically inhibit CES1 and CES2 (Shimizu et al., 2014), respectively. Vinblastine and eserine selectively inhibit AADAC but also showed moderate inhibition against CES2 (Shimizu et al., 2014). Simvastatin strongly inhibits CES1 and CES2 activities (Shimizu et al., 2014). Inhibitors (benzbromarone: 10 μM; BNPP: 1 mM; others: 20 μM) were preincubated with different enzyme sources for 5 min at 37°C, and reactions were initiated by adding vicagrel solution (1 μM). After 2 min of incubation, the reactions were terminated by adding equal volumes of ice-cold acetonitrile. Control samples were incubated with thermally inactivated enzymes.

### Contribution of CES2 and AADAC to Vicagrel Hydrolase Activity

The RAF method was applied as the ratio of activity values to evaluate the contribution of CES2 and AADAC to vicagrel hydrolysis in HIM. As described previously by Watanabe et al. (2010), the RAF value for CES2 (RAF_CES2, HIM_) was determined as the ratio of the hydrolase activities of the probe substrate procaine in HIM to the value of recombinant human CES2. Meanwhile, the RAF value for AADAC (RAF_AADAC, HIM_) was determined as the ratio of the hydrolase activities of the probe substrate phenacetin in HIM to the value of recombinant human AADAC. The following formulas were used to predict the vicagrel hydrolase activities of CES2 (*V*_CES2, HIM_), and AADAC (*V*_AADAC, HIM_) in HIM:

(2) $$V_{CES2,\;HIM}\;=\;V_{rec-CES2}\;\times \;RAF_{CES2,\;HIM}$$

(3) $$V_{AADAC,\;HIM}\;=\;V_{rec-AADAC}\;\times \;RAF_{AADAC,\;HIM}$$

V _rec-CES2_ and V _rec-AADAC_ are the vicagrel hydrolase activities of recombinant CES2 and AADAC, respectively. The following equations were used to calculate the contributions of CES2 and AADAC to vicagrel hydrolase activities in HIM:

(4) $$\text{CES2 in HIM}(\%)\;=\;(V_{CES2,\;HIM}/V_{HIM})\;\times \;100$$

(5) $$\text{AADAC in HIM}(\%)\;=\;(V_{AADAC,\;HIM}/V_{HIM})\;\times \;100$$

where the V _HIM_ value is the observed vicagrel hydrolase activities in HIM.

The RAF method can also be used to calculate the contributions of CES and AADAC in the liver, and the CES1 marker substrate is fenofibrate. However, as vicagrel is completely hydrolyzed during intestinal absorption, the contribution of enzymes in the liver is of little significance. Thus, we mainly focused on esterases in human intestine.

### LC-MS/MS Bioanalytical Method

The calibration curve concentration ranged from 2.00 to 4000 nmol/L for 2-oxo-clopidogrel. The calibration curve was fitted using a linear least-squares regression model (*y* = 1/*x*^2^). Calibration samples were extracted alongside study samples. Aliquots (50 μL) of either the calibrant or the study samples were added to 1.5 mL polypropylene tubes. A total of 25 μL of internal standard solution (IS, 2-oxo-clopidogrel-d_3_, 30.0 ng/mL) was then added, followed by 150 μL acetonitrile to precipitate proteins. The mixture was vortexed and centrifuged at 11,000 × *g* for 5 min. Finally, the supernatant was diluted with water and injected into the LC-MS/MS system.

Liquid chromatography-tandem mass spectrometry data were acquired using an LC-30AD liquid chromatographic system (Shimadzu, Kyoto, Japan) coupled to a Triple Quad 5500 mass spectrometer (AB Sciex). Analyst V1.6.2 software (AB Sciex) was used for data processing. Chromatographic separation was conducted on a Phenomenex Luna 5u PFP (2) (50 mm × 2.0 mm I.D., 5 μm), which was maintained at 40°C. The mobile phases used for gradient elution were 50% (solvent A) 5 mM ammonium acetate–formic acid (100/0.1, v/v) and 50% (solvent B) methanol. The initial mobile phase was 50%B for 0.8 min, followed by a linear increase to 85% over 0.1 min, which was then maintained for 1.0 min. The column was equilibrated with the initial mobile phase. The total run time was 3.0 min, and the flow rate was 0.50 mL/min. 2-oxo-clopidogrel and IS exhibited peak retention times of 2.1 min. A mass spectrometer equipped with an electrospray ionization source was operated in positive multiple reaction monitoring mode. The ion spray voltage and source temperature were set to 5,500 V and 500°C, respectively. Nebulizer gas, heater gas, curtain gas, and collision-activated dissociation gas were optimized at 50, 50, 30, and 9 psi, respectively. Samples were detected using multiple reaction monitoring, and the parent-to-product transitions were as follows: 2-oxo-clopidogrel *m/z* 338.2→155.1, CE 38 eV; 2-oxo-clopidogrel-d_3_ *m/z* 343.2→160.1, CE 38 eV; H4-MP *m/z* 504.2→155.0, CE 60 eV; H4-d_3_-MP *m/z* 509.2→160.1, CE 60 eV; fenofibrate hydrolysis metabolite *m/z* 319.1→233.0, CE 30 eV; procaine hydrolysis metabolite *m/z* 138.1→120.0, CE 17 eV; and phenacetin hydrolysis metabolite *p*-phenetidine *m/z* 138.1→110.1, CE 21 eV.

## Results

### Kinetics of Vicagrel Hydrolysis

Liu et al. (2017) reported that vicagrel was completely hydrolyzed to form 2-oxo-clopidogrel through intestinal absorption after oral administration of vicagrel tablets to healthy subjects. The parent drug was undetectable in human plasma during the sampling period (Liu et al., 2017). The hydrolytic metabolite 2-oxo-clopidogrel that is absorbed into the body is metabolized to the endo form by paraoxonase in plasma (Dansette et al., 2012; Qiu et al., 2016), used to produce the active metabolite H4 by CYP450, or hydrolyzed to carboxylic acid metabolites by liver esterases (Zhu et al., 2013). Therefore, the first hydrolysis step to produce 2-oxo-clopidogrel is very important for its bioactivation *in vivo*.

**FIGURE 2:**
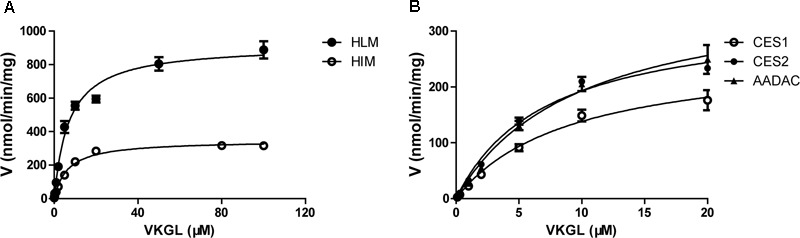
Kinetic analyses of vicagrel hydrolase activity by HLM and HIM **(A)** and recombinant CES1, CES2, and AADAC **(B)**.

For kinetic analyses of vicagrel hydrolase activity, probe substrates, including fenofibrate, procaine, and phenacetin were used as markers of CES1, CES2, and AADAC, respectively, to confirm the hydrolase activities of these enzyme sources. The hydrolytic rates of the three substrates were 213, 4.01, and 0.102 nmol/min/mg protein in HLM, respectively; in HIM, the hydrolytic rates of procaine and phenacetin were 2.72 and 0.094 nmol/min/mg protein, respectively (**Table 2**). The hydrolysis activity of vicagrel to form 2-oxo-clopidogrel was measured in all recombinant human esterases, and data for these activities followed the Michaelis–Menten equations (**Figure 2**). The *K*_m_, *V*_max_, and CL_int_ values of vicagrel hydrolysis in HIM were 6.54 ± 0.45 μM, 347.2 ± 6.4 nmol/min/mg protein, and 53.1 ± 1.0 mL/min/mg protein, respectively. Human CES2 and AADAC are expressed in gastrointestinal epithelial cells, and CES2 has been reported to catalyze the formation of 2-oxo-clopidogrel (Qiu et al., 2016). However, this is the first study to report that human AADAC is involved in vicagrel hydrolysis. The *K*_m_-values of vicagrel hydrolysis by CES2 and AADAC were 7.19 ± 0.16 and 9.79 ± 1.35 μM, respectively. The CL_int_ values were 46.1 ± 3.1 and 39.0 ± 3.1 mL/min/mg protein, respectively, indicating that the enzyme affinities were comparable.

**FIGURE 3:**
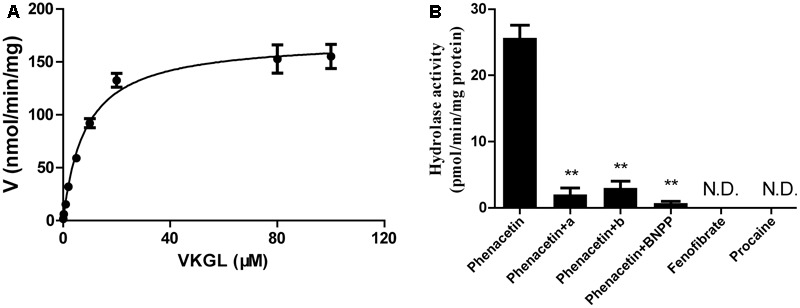
Kinetic analyses of vicagrel to form 2-oxo-clopidogrel in DIM **(A)**, and hydrolase activity of fenofibrate, procaine, and phenacetin in DIM **(B)**. Compound a and b were selective AADAC inhibitor vinblastine (10 μM) and eserine (10 μM), respectively. ^∗∗^*p* < 0.01.

We also investigated the hydrolysis of vicagrel in DIM (**Figure 3A**). The kinetic parameters are shown in **Table 1**. The CL_int_ value in DIM was 20.5 ± 1.3 mL/min/mg protein, which was lower than that in HIM. The *K*_m_-value was comparable among HIM, CES2, and AADAC (**Table 1**). Only phenacetin could be hydrolyzed (**Figure 3B**) after incubation separately of phenacetin, procaine and phenacetin in DIM, indicating that DIM have as considerable amount of AADAC activity but do not have any CES activity.

**Table 1:** Kinetic parameters of vicagrel hydrolysis by recombinant esterases and human tissue microsomes.

Enzyme	*K*_m_	*V*_max_	CL_int_
Source	μM	nmol/min/mg protein	mL/min/mg protein
HLM	7.18 ± 0.66	917.6 ± 23.5	127.8 ± 9.4
HIM	6.54 ± 0.45	347.2 ± 6.4	53.1 ± 1.04
CES1	9.12 ± 1.25	264.0 ± 16.6	29.0 ± 2.3
CES2	7.19 ± 0.16	331.2 ± 14.9	46.1 ± 3.1
AADAC	9.79 ± 1.35	381.7 ± 24.7	39.0 ± 3.1
DIM	8.37 ± 0.69	171.4 ± 3.9	20.5 ± 1.3

### Contribution of CES2 and AADAC to Vicagrel First-Pass Hydrolysis

To investigate the contribution of each esterase to the hydrolysis of vicagrel in the first-pass metabolism in the intestine, the contributions of CES2 and AADAC to the hydrolysis process were evaluated according to the previously reported RAF method (Watanabe et al., 2010). In HIM and CES2, the procaine hydrolase activity values were 2.72 and 3.34 nmol/min/mg protein, respectively, and the calculated RAF_CES2, HI_ value was 0.814. In HIM and AADAC, the phenacetin hydrolase activity values were 94.0 and 80.4 pmol/min/mg protein, respectively, and the calculated RAF_AADAC, HIM_ value was 1.17. The observed V_rec-CES2_ and V_rec-AADAC_ values were 26.1 and 21.8 nmol/min/mg protein, respectively. Thus, by using Equations (2) and (3), the calculated V_CES2, HIM_ and V_AADAC, HIM_ values were 21.2 and 25.2 nmol/min/mg protein, respectively. Finally, the contribution ratios were estimated according to Equations (4) and (5), resulting in CES2 and AADAC values of 44.2 and 53.1%, respectively (**Table 2**); the sum of the contributions from these two enzymes was 97.3%.

**Table 2:** Relative activity factor values calculated from the marker activity and the contributions of, CES2 and AADAC to vicagrel hydrolysis in HIM.

Enzyme source	Hydrolase activity				RAF			Contribution		
	Fenofibrate	Procaine	Phenacetin	Vicagrel	CES1	CES2	AADAC	CES1	CES2	AADAC
	nmol/min/mg	nmol/min/mg	pmol/min/mg	nmol/min/mg	%	%	%	%	%	%
CES1	92.8			17.3						
CES2		3.34		26.1						
AADAC			80.4	21.8						
HLM	213	4.01	102	96.5	2.30	1.20	1.27	-	-	-
HIM	0	2.72	94	48.0	0	0.814	1.17	-	44.2	53.1

### Inhibition Studies on Vicagrel Hydrolase Activity

**FIGURE 4:**
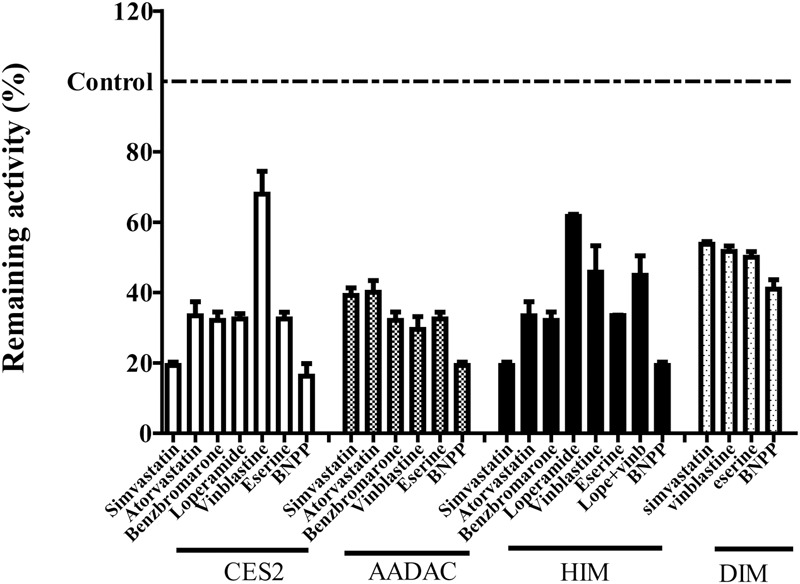
Inhibitory profile of chemical inhibitors on vicagrel hydrolase activity. BNPP (1 mM) is a non-selective inhibitor of esterases; loperamide (20 μM) is a specific inhibitor of CES2; vinblastine and eserine (both 20 μM) are selective inhibitors of AADAC; simvastatin, atorvastatin, and benzbromarone are strong inhibitors of CES and AADAC.

To evaluate the contribution of each esterase to vicagrel hydrolysis in human intestine, we investigated the effects of various chemical inhibitors on vicagrel hydrolase activity (**Figure 4**). The non-specific esterase inhibitor BNPP efficiently inhibited vicagrel hydrolysis by different enzyme sources. In HIM, 20 μM loperamide only inhibited hydrolysis by 40%, indicating the presence of enzymes other than CES2 that could hydrolyze vicagrel.

**FIGURE 5:**
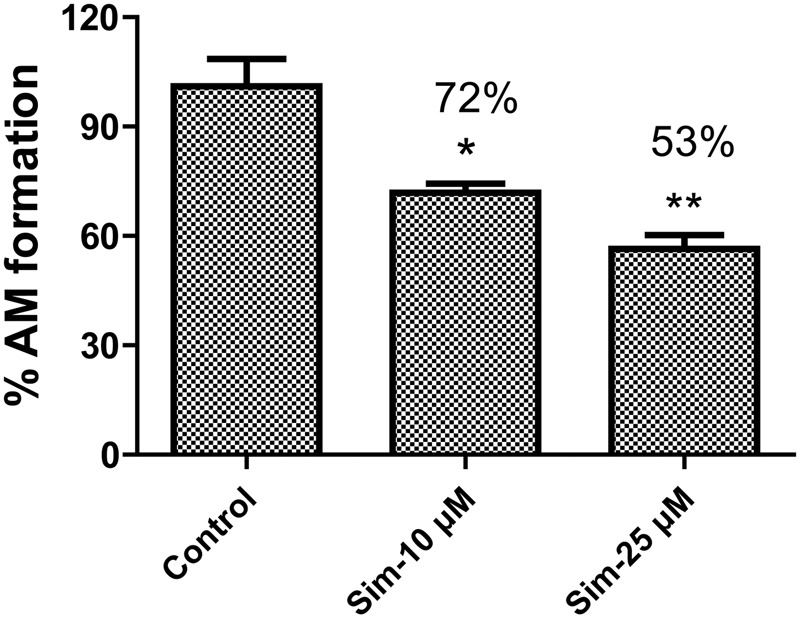
Simvastatin inhibited the production of active metabolites (AM) from vicagrel in HIM, with the concentration at 10 and 50 μM, respectively. ^∗^*p* < 0.05, ^∗∗^*p* < 0.01.

Statins (especially simvastatin) are often used in combination with clopidogrel. Therefore, during the development of vicagrel, we should evaluate the influences on metabolism and pharmacokinetics of vicagrel after combination with simvastatin. Simvastatin strongly inhibited CES activity (Shimizu et al., 2014; Wang et al., 2015), which significantly inhibited vicagrel hydrolysis in various enzymes at the final concentration of 20 μM in this study. Moreover, another statin drug, atorvastatin, also showed an inhibition effect, but this effect was lower than that of simvastatin (**Figure 4**). Formation of the active metabolite H4 from vicagrel was moderately inhibited by simvastatin; H4 production was reduced by 30% by 10 μM simvastatin (**Figure 5**). The AADAC-selective inhibitors vinblastine and eserine strongly inhibited vicagrel hydrolysis, indicating the involvement of AADAC in vicagrel hydrolysis in human intestine.

Dog intestine contained AADAC activity but did not contain CES activity (**Figure 3**). The addition of BNPP, simvastatin, vinblastine, and eserine to the incubation system of DIM significantly inhibited the hydrolysis of vicagrel (**Figure 4**), indicating that AADAC is the main enzyme responsible for vicagrel hydrolysis in dogs.

## Discussion

Vicagrel was designed to avoid metabolism by liver CYP2C19 and to be metabolized instead by esterases in the intestine and liver, aiming to produce H4 more efficiently and consistently in humans than clopidogrel. The most important intermediate metabolite produced from vicagrel, 2-oxo-clopidogrel, is produced by esterase hydrolysis during the gastrointestinal tract absorption process and then undergoes extensive metabolism in the body. Some amount of 2-oxo-clopidogrel produce active thiol metabolites in the presence of enzymes such as CYP3A4, CYP2B6, CYP2C9 and/or CYP2C19 in the intestine or liver (Kazui et al., 2010); some 2-oxo-clopidogrel in human or dog plasma form inactive endo form metabolites in the presence of paraoxonase (Qiu et al., 2016); and some produce inactive carboxylic acid metabolites (Zhu et al., 2013) (**Figure 1**). Vicagrel undergoes complete first-pass metabolism to produce 2-oxo-clopidogrel through intestinal absorption after oral administration of vicagrel tablets in healthy subjects; the parent drug was undetectable in human plasma (Liu et al., 2017). Hence, elucidation of the esterases involved in the first hydrolytic step is of great importance. Some reports have stated that CES2 is responsible for vicagrel hydrolysis in the intestine (Qiu et al., 2014, 2016), but this cannot explain the efficient hydrolysis of vicagrel in dog intestine, because no CES activity is present in dog intestine. This prompted us to find another esterase that possibly participates in vicagrel hydrolysis.

It was discovered that AADAC is involved in the bioactivation of vicagrel in the first-pass metabolism in human gastrointestinal system to produce 2-oxo-clopidogrel. The enzymatic kinetics of vicagrel in HIM and recombinant CES2 and AADAC were investigated *in vitro*; the *K*_m_-values were comparable in these enzyme sources, indicating that the enzyme affinities were similar. The CL_int_ values were 46.1 ± 3.1 and 39.0 ± 3.1 mL/min/mg protein in CES2 and AADAC, respectively. The contributions of CES2 and AADAC to the hydrolysis of vicagrel were calculated by the RAF method using procaine and phenacetin as CES2 and AADAC specific probe substrates; the values were 44.2 and 53.1%, respectively. In addition to CES2, intestinal AADAC was also involved in vicagrel hydrolysis before it reached the systemic circulation and therefore, plays an important role in vicagrel biological activation.

We performed inhibition studies with chemical inhibitors to confirm the involvement of AADAC in the hydrolysis of vicagrel. The AADAC-selective inhibitors vinblastine and eserine significantly inhibited the formation of 2-oxo-clopidogrel (**Figure 4**); the hypoglycaemic agent simvastatin not only inhibited vicagrel hydrolysis but also inhibited the production of the active metabolite H4, which suggests that clinical attention should be paid to the therapeutic efficacy and side effects of vicagrel when combined with simvastatin.

The hydrolytic rate of the AADAC-specific substrate phenacetin in DIM was approximately 30 pmol/min/mg protein (**Figure 4**); the clearance of vicagrel was 20.5 ± 1.3 mL/min/mg protein (**Table 1**). Dog intestine contains considerable AADAC activity (Kurokawa et al., 2016), and AADAC is the main esterase for vicagrel hydrolysis in dog intestine.

The production of the active metabolite H4 from vicagrel was four to six times higher than that of clopidogrel after oral administration of the same dose to rats and dogs. Comparable antiplatelet effects were observed with an oral dose of 5 mg vicagrel and 75 mg clopidogrel in healthy subjects (Liu et al., 2017), demonstrating the advantages of vicagrel as a new antiplatelet drug. This result is due to the difference in the first activation step for vicagrel and clopidogrel. The first step in the metabolic activation of vicagrel is the generation of 2-oxo-clopidogrel by human intestine CES2 and AADAC, which is complete and rapid, followed by oxidation by intestine and liver CYP450s to produce the active thiol metabolite H4. However, for clopidogrel, formation of active H4 requires two steps of CYP450 catalysis, producing 2-oxo-clopidogrel first and then H4. By comparing the two activation processes, we found that the formation of 2-oxo-clopidogrel from vicagrel was rapid and complete and that H4 had been produced before entering the systemic circulation, suggesting a shorter onset time. Moreover, CES2 and AADAC rather than CYP450, catalyzed the formation of 2-oxo-clopidogrel from vicagrel, suggesting that vicagrel was not affected by “clopidogrel resistance” due to CYP450 (mainly CYP2C19) gene polymorphisms.

This study is the first to report that AADAC in the human intestine is involved in the first-pass metabolism of vicagrel with a contribution of approximately 53%. Vicagrel undergoes complete hydrolytic metabolism to produce 2-oxo-clopidogrel during gastrointestinal absorption, and the active metabolite H4 is subsequently produced by intestinal and hepatic CYP450s. The production efficiency of active H4 was higher than that of clopidogrel in human pharmacokinetics. This study deepens the understanding of the bioactivation and metabolism properties of vicagrel in humans, which can help further elucidate the bioactivation mechanism of vicagrel and the variations in the treatment responses to vicagrel and clopidogrel.

## Author Contributions

JJ and DZ are responsible for the research design. JJ conducted experiments. DZ and XC contributed new reagents or analytical tools. JJ and DZ performed data analysis and wrote this manuscript.

## Conflict of Interest Statement

The authors declare that the research was conducted in the absence of any commercial or financial relationships that could be construed as a potential conflict of interest.

## Abbreviations

AADAC: arylacetamide deacetylase
BNPP: bis-*p*-nitrophenyl phosphate
CES1: carboxylesterase 1
CES2: carboxylesterase 2
DIM: dog intestine microsomes
HIM: human intestine microsomes
HLM: human liver microsomes
LC-MS/MS: liquid chromatography-tandem mass spectrometry
RAF: relative activity factor
VKGL: vicagrel
